# Zinc Supplementation with or without Additional Micronutrients Does Not Affect Peripheral Blood Gene Expression or Serum Cytokine Level in Bangladeshi Children

**DOI:** 10.3390/nu13103516

**Published:** 2021-10-07

**Authors:** Thomas Hayman, Peter Hickey, Daniela Amann-Zalcenstein, Cavan Bennett, Ricardo Ataide, Rahvia Alam Sthity, Afsana Mim Khandaker, Kazi Munisul Islam, Katharina Stracke, Nawaf Yassi, Rosie Watson, Julie Long, Jamie Westcott, Nancy F. Krebs, Janet C. King, Robert E. Black, Md. Munirul Islam, Christine M. McDonald, Sant-Rayn Pasricha

**Affiliations:** 1 Population Health and Immunity Division, Walter and Eliza Hall Institute of Medical Research, Parkville, VIC 3052, Australia; hayman.t@wehi.edu.au (T.H.); bennett.c@wehi.edu.au (C.B.); ataide.t@wehi.edu.au (R.A.); stracke.k@wehi.edu.au (K.S.); yassi.n@wehi.edu.au (N.Y.); watson.r@wehi.edu.au (R.W.); 2 Department of Medical Biology, University of Melbourne, Parkville, VIC 3010, Australia; hickey@wehi.edu.au (P.H.); zalcenstein.d@wehi.edu.au (D.A.-Z.); 3 Advanced Technology and Biology Division, Walter and Eliza Hall Institute of Medical Research, Parkville, VIC 3052, Australia; 4 International Centre for Diarrheal Disease Research, Nutrition and Clinical Services Division, Bangladesh (icddr,b), Dhaka 1212, Bangladesh; rahviaalam@gmail.com (R.A.S.); akhandaker@nevada.unr.edu (A.M.K.); munisul@icddrb.org (K.M.I.); mislam@icddrb.org (M.M.I.); 5 Melbourne Brain Centre, Departments of Medicine and Neurology, The Royal Melbourne Hospital, University of Melbourne, Parkville, VIC 3050, Australia; 6 Department of Medicine, The Royal Melbourne Hospital, University of Melbourne, Parkville, VIC 3050, Australia; 7 Department of Pediatrics, Anschutz Medical Campus, University of Colorado, Aurora, CO 80045, USA; julie.long@cuanschutz.edu (J.L.); Jamie.Westcott@CUAnschutz.edu (J.W.); Nancy.Krebs@CUAnschutz.edu (N.F.K.); 8 International Zinc Nutrition Consultative Group, University of California, San Francisco, CA 94158, USA; jking829@berkeley.edu (J.C.K.); rblack1@jhu.edu (R.E.B.); Christine.mcdonald@ucsf.edu (C.M.M.); 9 Department of Nutritional Sciences and Toxicology, University of California, Berkeley, CA 94720, USA; 10 Institute for International Programs, Bloomberg School of Public Health, Johns Hopkins University, Baltimore, MD 21218, USA; 11 Departments of Pediatrics and Epidemiology and Biostatistics, School of Medicine, University of California, San Francisco, CA 94158, USA; 12 Diagnostic Haematology, The Royal Melbourne Hospital, Parkville, VIC 3050, Australia; 13 Clinical Haematology at the Peter MacCallum Cancer Centre, The Royal Melbourne Hospital, Parkville, VIC 3050, Australia

**Keywords:** zinc, RNA sequencing, Bangladesh, transcriptomics, immunology

## Abstract

Preventive zinc supplementation provided as a stand-alone dispersible tablet, or via home fortification as multiple micronutrient powders (MNPs), has been considered a potential strategy to prevent zinc deficiency and improve health (including immune) outcomes among children in low- and middle-income countries. However, the impact of zinc supplementation on immune profiles has not been well characterized. We sought to define the effect of zinc supplementation on peripheral blood gene expression and cytokine levels among young children in Dhaka, Bangladesh. In a sub-study of a large randomized, controlled, community-based efficacy trial where children 9–11 months of age received one of the following interventions on a daily basis for 24 weeks: (1) MNPs containing 10 mg of zinc; (2) dispersible tablet containing 10 mg zinc; or (3) placebo powder, we used RNA sequencing to profile the peripheral blood gene expression, as well as highly sensitive multiplex assays to detect cytokine profiles. We profiled samples from 100 children enrolled in the parent trial (zinc MNPs 28, zinc tablets 39, placebo 33). We did not detect an effect from either zinc intervention on differential peripheral blood gene expression at the end of the intervention, or an effect from the intervention on changes in gene expression from baseline. We also did not detect an effect from either intervention on cytokine concentrations. Exploratory analysis did not identify an association between undernutrition (defined as stunting, underweight or wasting) and peripheral blood gene expression. Zinc interventions in children did not produce a gene expression or cytokine signature in the peripheral blood. However, this study demonstrates a proof of principle that sensitive multi-omic techniques can be applied to samples collected in field studies.

## 1. Introduction

Globally, over 17% of the world’s population has been considered at risk of inadequate nutritional zinc intake [1], and zinc deficiency is especially widespread across low- and middle-income countries [2]. Zinc deficiency has been linked to impaired child growth and is common in children living in settings where dietary diversity and consumption of animal-source foods are limited [1]. Therapeutic zinc supplementation reduces the duration of diarrhea in children [3] and is part of the World Health Organization (WHO) Integrated Management of Childhood Illness guidelines for treatment of diarrhea [4]. Preventive zinc supplementation provided as a stand-alone dispersible tablet, or via home fortification as multiple micronutrient powders (MNPs), has been considered a potential strategy to prevent zinc deficiency in low- and middle-income countries (LMICs). Such an approach could improve functional outcomes including reduced diarrhea, improved child growth and lower mortality [5,6].

Zinc is an essential trace element that is crucial to cellular metabolism: it is critical to the function of numerous transcription factors and is involved in the activity of numerous enzymes and cellular functions [7]. Experimental exposure of lymphocyte cell lines to supplemental zinc promotes the expression of thousands of genes. Zinc deficiency may promote a pro-inflammatory phenotype [8]. Systemic zinc administration has thus been thought to improve host immune function. However, the impact of preventive zinc supplementation in children on immune function has not been clearly defined experimentally. Whether zinc administration mediates its beneficial effects on clinical health via an effect on immune function has not been determined. 

RNA sequencing is an established technique for measuring levels of mRNA expressed in a particular tissue or sample. The technique utilizes next-generation sequencing to measure the presence and abundance of all transcripts in a sample (‘transcriptome’) [9]. This technique is becoming increasingly valuable for discovering new mechanisms and biomarkers associated with interventions and outcomes in samples collected from clinical studies. In addition, there are increasingly sensitive techniques for measuring the abundance of cytokines and other circulating proteins in serum or plasma. 

We sought to leverage these technologies to define the effect of preventive zinc supplementation on cellular gene expression and cytokine levels. Furthermore, we sought to assess whether peripheral blood gene expression could be used as a biomarker for zinc intervention status. In a sub-study of young children enrolled in a large randomized, controlled, community-based efficacy trial in Dhaka, Bangladesh [10], we undertook transcriptome-wide RNA sequencing and high-sensitivity cytokine analysis to define the effects of preventive zinc supplementation (provided as either a daily dispersible tablet or as a component of MNPs), in comparison with placebo powders, on peripheral blood gene expression and cytokine levels. 

## 2. Materials and Methods

### 2.1. Parent Trial

The present analysis is a sub-study of a randomized, controlled, partially blind, community-based efficacy trial comparing five different doses, frequencies and/or forms of preventive zinc supplementation to a placebo powder, provided for 24 weeks to children 9 to 11 months of age living in Dhaka, Bangladesh (clinicaltrials.gov NCT03406793) [10]. The primary outcomes of the parent trial were incidence of diarrhea and linear growth (change in length-for-age z-score (LAZ)), measured and analyzed according to the 2006 WHO Child Growth Standards [11]. Children were eligible for the trial if they met the age inclusion criteria and did not have evidence of severe acute malnutrition, severe anemia, congenital abnormalities or other serious chromosomal or medical conditions. The six-arm trial consisted of three different active daily micronutrient powders containing 15 micronutrients including (i) 4.1 mg zinc and 10 mg iron (i.e., standard MNP formulation); (ii) 10 mg zinc and 6 mg iron; and (iii) 10 mg zinc and 6 mg iron alternating with 10 mg zinc and 0 mg iron; dispersible zinc (10 mg) tablets administered either (iv) daily or (v) daily for 14 days immediately at enrollment and after 12 weeks of follow-up with daily placebo tablets in the intervening periods; and (vi) daily placebo powders, all administered orally. In the arms utilizing MNPs, the dose was mixed into the first spoonfulls of food. Blood samples for the analyses reported in the present study were obtained from children in three arms: those receiving the MNPs containing 10 mg zinc and 6 mg iron (high-zinc, low-iron MNPs), daily dispersible 10 mg zinc tablets (zinc tablets) and placebo powder. We selected these arms as they represented the two groups with the highest zinc dosing, along with placebo. 

### 2.2. Sample Collection and Shipping 

Blood samples were collected from participants in the subgroup at enrolment and after the completion of the 24-week intervention period. Blood was collected in both an EDTA and clot activator (serum) tube. RNA stabilization was achieved as follows: 0.5 mL of the EDTA sample was placed into a cryovial containing 1.3 mL of RNA*later*^TM^; the tube was inverted 4–5 times and kept on ice until it could be placed into a −80 °C freezer for storage, usually within 24 to 48 h. Up to 1 mL of serum was aliquoted and stored at −80 °C. Serum and whole blood samples were transported from Dhaka to Melbourne, Australia, on dry ice. Scientists remained blinded to the trial allocation arms until after experimental and bioinformatic analysis was complete.

### 2.3. RNA Extraction

Frozen samples were thawed at room temperature, and RNA was isolated immediately using the RiboPure Blood Kit (Ambion) as per the manufacturer’s protocol. After extraction, the concentration and purity of RNA were quantified by measuring the absorbance at 260 nm (A260) and 280 nm (A280) using a NanoDrop ND-100 spectrophotometer (Thermo Scientific, Waltham, MA, USA). Eluted RNA was stored at −80 °C until experimental analysis. 

### 2.4. Library Preparation and RNA Sequencing

RNA was thawed on ice before being normalized to 50 ng/μL in order to obtain uniform library sizes. Bulk gene expression with RNA sequencing was performed on whole blood samples from the two intervention groups and placebo at baseline and endline to assess the molecular effect of zinc supplementation on peripheral blood gene expression, and to probe for a possible biomarker for zinc supplementation status. Transcriptome libraries were generated by adapting the CelsSeq2 protocol [12] as follows: Samples were pooled after first-strand cDNA synthesis and treated with Exonuclease 1 for 30 min, followed by a 1.2X bead clean-up. Second-strand synthesis was performed using the NEBNext Second Strand Synthesis module (NEB #E6111S) in a final reaction volume of 20 μL, and NucleoMag NGS Clean-up and Size select magnetic beads (Macherey-Nagel—7449970.5) were used for all DNA purification and size selection steps.

### 2.5. Bioinformatic Analysis of RNA Sequencing

RNA sequencing reads were mapped to the GRCh38.p12 human genome and ERCC spike-in sequences using the Subread aligner (v2.2.6) [13] and assigned to genes using scPipe (v1.10.0, WEHI, Parkville, VIC, Australia) [14] with GENCODE v28 primary assembly annotation. Gene counts were exported as a matrix by scPipe with UMI-aware counting. All subsequent analysis was performed in R (version 4.0.0, R-Core team, Vienna, Austria) [15] with Bioconductor (version 3.11).

Samples were removed from further analysis if they failed to achieve QC cutoffs for total UMI counts and total genes detected. Overall, 4 samples (technical duplicates of 2 samples) were excluded, leaving 388 samples (technical duplicates of 194 samples) for downstream analysis. All genes detected in at least 1 sample were used for downstream analysis (34,713 genes). The ‘filterByExpr’ function in edgeR (v3.30.3, WEHI, Parkville, VIC, Australia) was used to determine which genes had sufficiently large counts to be retained in a differential expression analysis. Specifically, 5778 genes with a count per million (CPM) of at least 60.2 (about 10 UMI counts) in at least 56 samples (the minimum experimental group size) were retained. The counts were filtered and normalized by the method of trimmed mean of M-values (TMM). Data were visualized and normalized by performing multi-dimensional scaling (MDS) of the log2 counts per million (logCPM) values for the top 500 genes using the ‘plotMDS’ function in edgeR (v3.30.3). As the experiment was fully replicated across plates, the design matrix was used to remove the effect of plate-to-plate differences on downstream analyses. 

### 2.6. RNA Sequencing Analyses

Our primary analysis aimed to identify genes that were differentially expressed between the different treatments and timepoints. Specifically, we looked at pairwise comparisons of treatments at endline; pairwise comparisons of timepoints within each treatment; and pairwise comparisons of the interaction between timepoint and treatment (i.e., pairwise comparisons of deltas). Our secondary analysis aimed to determine which genes have their expression associated with poor growth, specifically stunting (length-for-age z-score < −2); underweight (weight-for-age z-score < −2); and wasting (weight-for-length z-score < −2). 

For the primary comparisons, we used an additive design matrix that included a term for plate number, experimental group (i.e., the combination of timepoint and treatment) and blocked on sample to enable estimation of the intra-sample correlation between the technical duplicates. For the secondary comparisons, we used only the endline samples and added a term for the relevant additional covariate to the design matrix.

To perform differential expression analysis, we used the ‘voomLmFit’ function in edgeR (v3.30.3) that adapts the limma voom method [16] to allow for loss of residual degrees of freedom (df) due to exact zero counts [17]. We treated the sample as a blocking variable to enable estimation of the intra-sample correlation of the technical duplicates via the ‘duplicateCorrelation’ function in limma (v3.44.3, WEHI, Parkville, VIC, Australia) [18]. The ‘voomLmFit’ pipeline, run with quantile normalization and the ‘eBayes’ function in limma (v3.44.3), was used to compute robust empirical Bayes statistics for differential expression [19]. All code used to perform data processing and the analysis report of the RNA-seq data are available from https://github.com/WEHISCORE/C084_Hayman_Pasricha (accessed on 2 September 2021).

### 2.7. Simoa Analysis of Serum Cytokine Levels

Simoa (Quanterix) technology uses a magnetic bead-based multiplex array that allows for ultrasensitive detection of low-level proteins. It is an automated, reproducible technique for precise biomarker measurement in clinical blood samples [20]. Serum samples stored at −80 °C were thawed at room temperature, mixed thoroughly, centrifuged (10,000× *g*) and diluted (1:4) before being analyzed. All endline serum samples were analyzed in duplicate using the Cytokine 6-plex assay panel 1 (Quanterix, Billerica, MA, USA) for IFN-γ, IL-6, IL-10, IL-12p70, IL-17A and TNFα as per the manufacturer’s instructions. Difference in endline cytokine levels between the zinc tablet, zinc MNP and placebo groups was assessed using one-way ANOVA with *p*-values < 0.05 considered statistically significant.

## 3. Results

**Figure 1:**
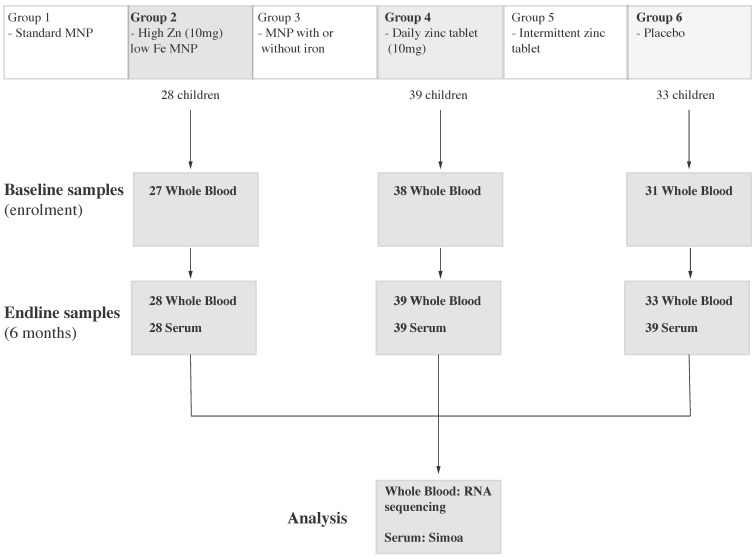
Study flow for the transcriptomic and immunologic evaluation of trial participants. Three study arms (Group 2, Group 4 and Group 6) were selected from the main ZIPT trial, reflecting high-dose zinc delivered as either micronutrient powders, dispersible tablets or placebo. The number of samples which were analyzed by RNA sequencing or Simoa is shown at each timepoint.

Whole blood and serum samples from 100 children enrolled in the parent trial underwent analysis for this sub-study. These included a total of 100 endline and 96 matched baseline whole blood samples, and 100 endline and 99 matched baseline serum samples from the zinc MNP, zinc tablet and placebo groups (Figure 1). 

Baseline characteristics of the participants, according to intervention group, are presented in Table 1. 

The average age of participants at recruitment was 9.7 months. Of the children in the sub-study, 49% were female. The prevalence of stunting (LAZ < −2) at baseline was 26%.

### 3.1. Neither Zinc MNPs nor Zinc Tablets Altered the Peripheral Blood Transcriptome 

**Figure 2:**
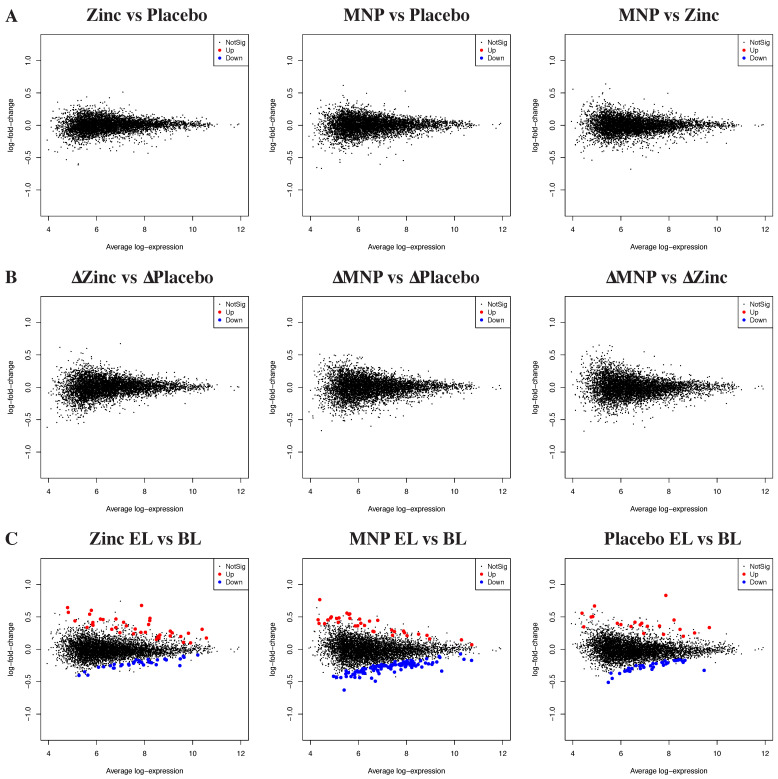
Comparative gene expression after zinc or MNP supplementation. RNA sequencing analysis of whole blood from participants treated with zinc tablets, MNPs or placebo powder showing (**A**) pairwise comparisons of differential gene expression between treatment arms at endline; (**B**) comparisons of the change in gene expression from baseline to endline between treatment arms; and (**C**) change in gene expression from baseline to endline within each treatment arm. Red = gene significantly upregulated. Blue = gene significantly downregulated. BL—baseline. EL—endline. Zinc—zinc 10 mg dispersible tablets; MNPs—14-micronutrient powders containing 10 mg zinc; placebo—placebo powders.

Transcriptomic data is available at the Gene Expression Omnibus (ID GSE184998; https://www.ncbi.nlm.nih.gov/geo/query/acc.cgi?acc=GSE184998 (accessed on 2 September 2021)). After quality control, 5788 genes had sufficient counts to perform differential expression analysis. Pairwise comparisons were conducted to compare the gene expression profiles from the zinc MNP and zinc tablet groups with placebo at endline. At the endline timepoint, no differences were observed between high-zinc, low-iron MNPs vs. placebo powder, between daily dispersible zinc tablets vs. placebo powder or between high-zinc, low-iron MNPs vs. daily dispersible zinc tablets (Figure 2A).

We then assessed the effect of zinc tablets or zinc MNPs on changes in gene expression from baseline to endline (delta, Δ) using matched baseline and endline samples. We observed no difference between baseline and endline in changes in gene expression between daily dispersible zinc tablets vs. placebo powders, high-zinc, low-iron MNPs vs. placebo powders or high-zinc, low-iron MNPs vs. daily dispersible zinc tablets (Figure 2B). A lack of differential gene expression and changes in differential gene expression between study groups was seen despite the substantial number of differentially expressed genes seen between the baseline and endline of the trial within each arm (Figure 2C). 

### 3.2. Differential Gene Expression by Growth Outcomes

**Figure 3:**
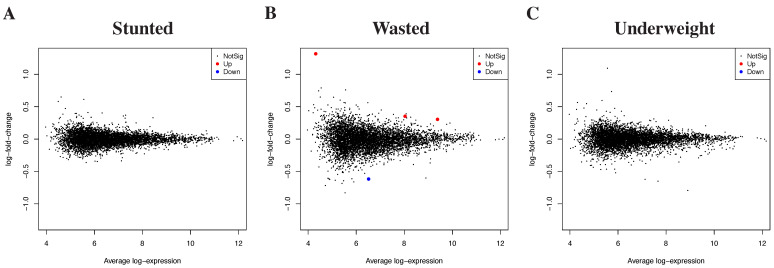
Differential gene expression at endline by growth outcomes. RNA sequencing analysis of whole blood from participants at endline, showing associations between gene expression and (**A**) stunting (length-for-age z-score < −2), (**B**) wasting (weight-for-length z-score < −2) and (**C**) underweight (weight-for-age z-score < −2). Red = gene significantly upregulated. Blue = gene significantly downregulated.

As a secondary analysis, the RNA sequencing dataset was used to explore associations between growth outcomes on peripheral blood gene expression (Figure 3). Specifically, we were interested in differentially expressed genes in children who were stunted, wasted or underweight. While no difference in gene expression was observed in the serum of stunted or underweight children, there were a small number of differentially expressed genes in wasted children compared to children without wasting: *YBX1P2*, *H3P6* and *H3P16* were upregulated (all three genes with unknown functions), and *STXBP5* (Syntaxin binding protein, involved in vesicle transport and neurotransmission) was downregulated (Benjamini–Hochberg adjusted *p* < 0.05).

### 3.3. Simoa Analysis of Serum Cytokine Levels

**Figure 4:**
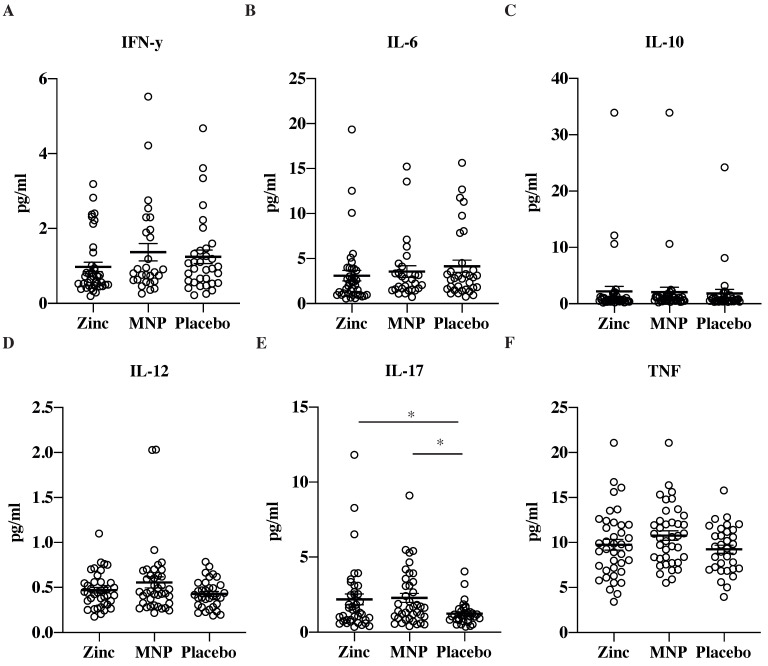
Analysis of serum cytokine levels after zinc tablet or multiple micronutrient powder (MNP) supplementation. Simoa multiplex analysis of serum samples taken from participants at endline: (**A**) IFN- γ, (**B**) IL-6, (**C)** IL-10, (**D**) IL-12, (**E**) IL-17 and (**F**) TNF after treatment with zinc (10 mg, in dispersible tablets), MNP (containing 10mg zinc) or placebo powder. * *p* < 0.05 by ANOVA.

We did not observe any significant difference between the high-zinc, low-iron MNP, daily dispersible zinc tablet and placebo powder groups in concentrations of IFN-γ, IL-6, IL-10, IL-12p70 and TNFα at endline (Figure 4). However, levels of IL-17A were higher in both the high-zinc, low-iron MNP and daily dispersible zinc tablet groups compared to the placebo powder group. Further interrogation of the RNA sequencing data likewise did not identify differential expression of these cytokine genes. 

## 4. Discussion

Preventive zinc supplementation has been explored as a potential public health intervention to improve zinc status, reduce diarrhea and improve growth in children. One hypothesized mechanism for this reduction has been the beneficial effects of routine zinc administration on immune function [8]. We measured transcriptome-wide peripheral blood gene expression and utilized ultrasensitive serum cytokine assays in a subgroup of Bangladeshi infants 9–11 months of age who participated in a randomized, controlled, community-based efficacy trial of different forms of zinc supplementation for the prevention of diarrhea and promotion of linear growth. We did not detect an effect from either supplemental zinc tablets or MNPs containing high-dose zinc (and low-dose iron) on peripheral blood gene expression or cytokine levels, nor could we detect a clear association between peripheral blood gene expression profiles and child growth. These results align with the clinical results of the parent trial, which did not detect any differences in the incidence or prevalence of diarrhea or related morbidity outcomes from active zinc interventions, demonstrating clear impacts on serum zinc concentrations.

Zinc has been considered essential for the normal development and function of a range of innate, cellular and adaptive immune processes [21]. Zinc is critically involved in the function of over 2000 transcription factors. For example, zinc depletion has been observed to activate the function of key innate immune-active signaling pathways such as NF-κB in in vitro models [22], while the zinc transporter ZIP8 is a negative regulator of NF-κB signaling [23]. Zinc deficiency may also impair lymphocyte differentiation, alter the ratio of Th1 and Th2 cells and modify interactions between T cells and dendritic cells, resulting in dysfunction of the adaptive immune system [8]. Zinc may potentiate T cell activation with enhanced IFN-γ [24]. Treatment of human monocyte/macrophage THP-1 cells with supplemental zinc or a zinc chelator produced differential expression of approximately 1000 genes (detected by microarray), including about 200 genes that were linearly altered as cellular zinc levels changed [25]. A previous analysis that applied transcriptome and cytokine analyses to samples collected from a cohort of previously healthy men subjected to dietary zinc depletion (0.3 mg zinc daily) found that depletion induced differential expression of 328 genes, with over-representation of genes involved in the cell cycle and cell-mediated immune responses [26].

The present study is among the first to utilize an unbiased transcriptome-wide approach to detect an effect of zinc supplementation in the peripheral blood, and to apply this approach to samples collected in a study population where zinc deficiency at baseline would be generally mild in severity. These previous human and experimental observations led us to hypothesize that treatment of children at high risk of zinc deficiency would likely induce detectable changes in peripheral blood gene expression or cytokine levels, which may indicate a systemic effect on peripheral immune function. The fact that we did not detect such an effect may be due to several reasons. Cellular zinc levels are highly buffered, and hence zinc depletion and restitution at a clinical level in this cohort may not have substantially altered intracellular zinc metabolism and function for cells involved in immune responses [27]. Our results are in line with a trial of preventive zinc supplementation in Laotian children, which likewise did not detect an effect from zinc on T cell cytokines, LPS-stimulated cytokines and T cell concentrations [28]. Previous data from in vitro treatment of cell lines with high or low zinc concentrations, from experimental animal models with markedly zinc-depleted or loaded diets or from humans receiving experimentally controlled zinc depletion diets may not apply to this more complex population of children with a variety of dietary and infectious exposures. For example, absorption and utilization of zinc in this population may be influenced by factors such as intestinal and systemic inflammation [29].

Our study utilized transcriptomic analysis on whole blood in order to discover a molecular biomarker for zinc repletion which could be subsequently adapted for simple use in field research and population studies. We were able to analyze the expression of about 5800 genes which had sufficient sequencing reads, likely related to expression in the cells contained in the whole blood sample. Differential gene expression among less abundant cell populations may have been masked using our approach of whole blood transcriptomics. Isolating peripheral blood mononuclear cells or even specific lymphocyte populations may have provided qualitatively different results [30] and could have allowed us to assay the effect of zinc supplementation on specific cell types. Such an analysis may have been more sensitive to zinc-induced differential gene expression in particular cellular compartments. We did identify a small number of genes that were differentially expressed in children with wasting; these may provide an opportunity for further understanding the mechanisms or effects of undernutrition in children, but validation of these findings across other populations is essential. 

Although we did not detect differentially expressed genes or cytokines in this analysis, our analyses indicate the experiments themselves were technically successful. Samples had been collected in the field in Dhaka, Bangladesh, and stabilized in the field laboratory. Our study demonstrates that cutting-edge transcriptomic and cytokine approaches can be applied to samples collected in field conditions in the future; such approaches will be integral to interrogating the biological correlates of public health interventions, and for discovering biomarkers associated with successful clinical endpoints. 

## 5. Conclusions

Zinc supplementation delivered as oral tablets or MNPs to Bangladeshi infants did not alter peripheral blood transcriptional profiles or cytokine levels. 

## Figures and Tables

**Table 1:** Characteristics of participants.

	Zinc MNPs	Zinc Tablets	Placebo	Difference Between Groups (*p*-Value, ANOVA)
N	28	39	33	
Age (months)	9.8 (0.94)	9.7 (0.87)	9.7 (0.85)	0.93
Sex N, (F%)	12 (42.9%)	20 (51.3%)	17 (51.5%)	0.75
WLZ at baseline	−0.66 (0.85)	−0.40 (0.88)	−0.61 (0.95)	0.85
WLZ after 24 weeks	−1.0 (0.87)	−0.68 (0.86)	−0.90 (0.94)	0.32
LAZ at baseline	−1.45 (0.84)	−1.23 (1.10)	−1.36 (1.08)	0.67
LAZ after 24 weeks	−1.70 (0.78)	−1.41 (1.10)	−1.50 (1.09)	0.52
WAZ at baseline	−1.29 (0.94)	−0.99 (1.08)	−1.22 (1.09)	0.42
WAZ after 24 weeks	−1.53 (0.91)	−1.16 (0.99)	−1.36 (1.01)	0.33
Any diarrhea within last 2 weeks at endline	1 (3.6%)	2 (5.1%)	2 (6.1%)	0.42

WLZ: weight-for-length z-score; LAZ: length-for-age z-score; WAZ: weight-for-age z-score. Results are presented as mean (SD) unless otherwise noted.

## Data Availability

Data will be available upon reasonable request from the authors. Please contact the corresponding author.
